# Improved infrared photoluminescence characteristics from circularly ordered self-assembled Ge islands

**DOI:** 10.1186/1556-276X-6-416

**Published:** 2011-06-09

**Authors:** Samaresh Das, Kaustuv Das, Raj Kumar Singha, Santanu Manna, Achintya Dhar, Samit Kumar Ray, Arup Kumar Raychaudhuri

**Affiliations:** 1 Department of Physics and Meteorology, Indian Institute of Technology Kharagpur, Kharagpur 721302, India; 2 DST Unit for Nanoscience, S N Bose National Centre for Basic Sciences, Block JD, Sector III, Kolkata 700098, India

## Abstract

The formation of circularly ordered Ge-islands on Si(001) has been achieved because of nonuniform strain field around the periphery of the holes patterned by focused ion beam in combination with a self-assembled growth using molecular beam epitaxy. The photoluminescence (PL) spectra obtained from patterned areas (i.e., ordered islands) show a significant signal enhancement, which sustained till 200 K, without any vertical stacking of islands. The origin of two activation energies in temperature-dependent PL spectra of the ordered islands has been explained in detail.

## Introduction

The confinement of charge carriers in low-dimensional Ge/Si heterostructures allows one to increase the efficiency of the radiative recombination, making the indirect gap group-IV semiconductors attractive for optical devices. Owing to the type-II band alignment [1], Ge dots form a potential well only for holes, whereas the electrons are weakly confined in the vicinity of the Ge dots, i.e., by the tensile strain field in the Si cap induced by Ge quantum dots (QDs) [2,3]. The resulting recombination energy depends strongly on size, shape, strain, and composition of the QDs leading to a wide emission energy spectrum. Therefore, intensive effort is currently undertaken to prepare arrays of "identical" QDs, which emit in a resonant mode [4]. Infrared (IR) photoluminescence (PL) at room temperature has been reported by vertical ordering of Ge islands in three-dimensional stack of 10-20 periods [5,6].

To improve the lateral ordering of QDs, one of the strategies is to convert the stochastic nucleation process into a deterministic one by directing nucleation on the predefined surface sites, using a combination of self-assembly and surface pre-patterning [7-10]. In general, the 2D Ge dot arrays reported so far have considerably larger inter-dot distance, thus lateral coupling is quite weak. The IR PL emission from randomly distributed islands is reported to be quenched at a relatively low temperature [2,11], because of thermal dissociation of excitons. In this article, we report the superior IR PL characteristics, which exist up to a temperature as high as 200 K, owing to lateral coupling in circularly ordered Ge islands on pre-patterned Si (001) substrates.

### Experimental

Ge QDs were grown by solid source molecular beam epitaxy (MBE) on focused ion beam (FIB) patterned (FEI HELIOS 600 dual beam system) substrates. The Si (001) substrate surface was patterned with two-dimensional periodic hole arrays using an FIB with Ga^+ ^ion energy of 30 keV and a beam current of 21 pA. Arrays of about 50 × 50 holes of diameter in the range of 100-200 nm and depth varying from 20 to 50 nm were fabricated at a fixed volume per dose (0.15 μm^3^/nC). The hole spacing and pitch were varied from 50 to nearly 200 nm and 50 to 600 nm, respectively. After removing Ga contamination from the surface, Ge QDs were grown using solid source MBE (Riber Supra 32) system using an electron gun for the deposition of thin buffer layer (approx. 5 nm) of Si with a growth rate of 0.4 Å/s, and a Knudsen cell for Ge deposition followed by a 2-nm Si cap layer. The Ge growth rate was kept constant at 0.5 Å/s at a substrate temperature of 580°C. PL spectra were recorded under excitation from a 325-nm He-Cd laser line with an output power of 1.3 W/cm^2 ^using a standard lock-in technique and a liquid N_2 _cooled InGaAs detector with a spectral range of 0.9-2.1 μm. The laser beam with a spot size of less than 500 μm was used for the selective probing of the sample in the patterned region.

## Results and discussion

**Figure 1:**
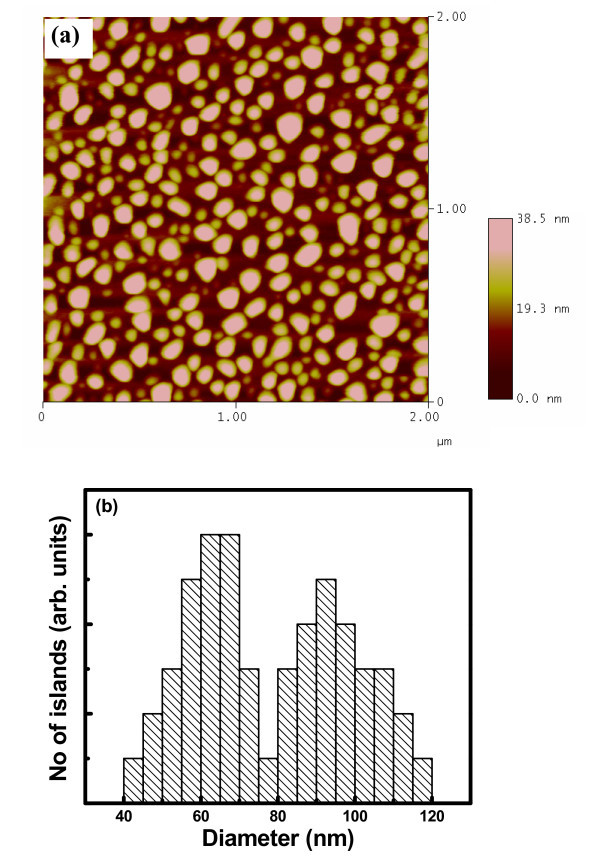
**(a) AFM image and (b) size distribution**.

**Figure 2:**
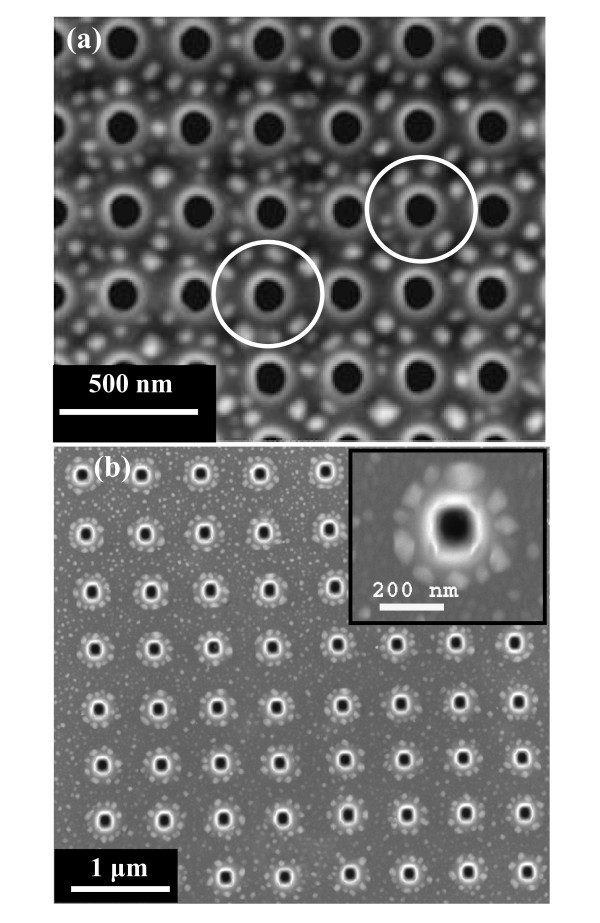
**SEM images of Ge islands grown on a FIB pre-patterned region with (a) smaller (approx. 160 nm) and (b) larger (approx. 500 nm) spacing of holes**. The circles drawn in Figure 1a show the ordering of islands along the circular periphery. The inset in (b) shows the array of islands in higher magnification.

**Figure 3:**
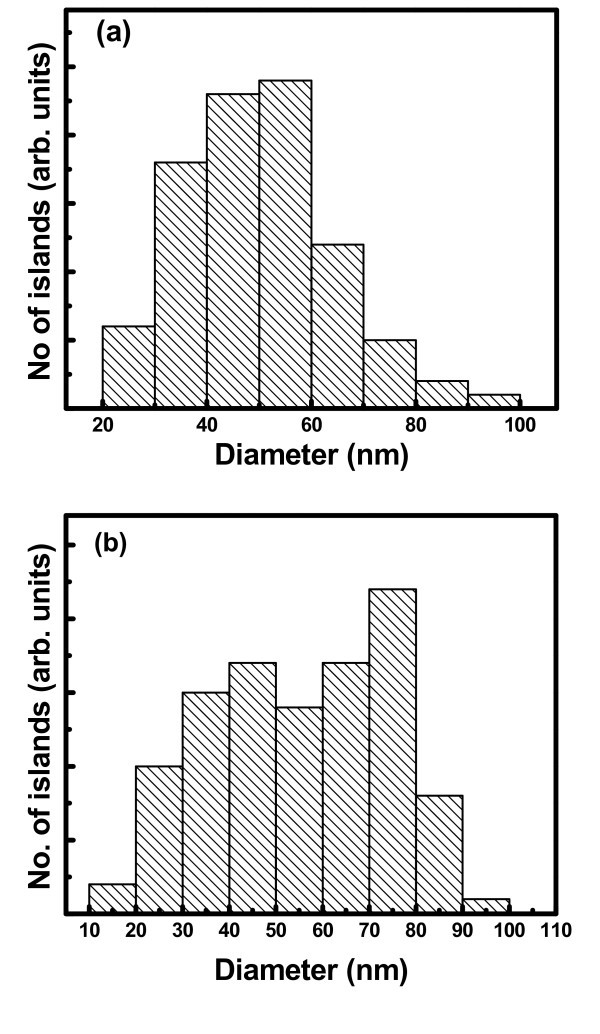
**The size distribution of Ge islands grown on patterned substrate with (a) 160-nm pitch and (b) 500-nm pitch**.

Microscopic analysis has been carried out in patterned as well as the unpatterned substrates to compare the nature of growth of Ge nanoislands. These experiments have been primarily done at different alloy compositions and growth conditions, where previous studies [11,12] have shown that it is possible to constrain island growth to occur only at the energetically favored edges. Figure 1a shows the atomic force microscopy (AFM) image of the unpatterned regions. From Figure 1b, it is clear that islands distribution is nearly bimodal in unpatterned area. The smaller islands have an average diameter of approx. 65 nm and height approx. 7 nm, whereas the larger ones are approx. 95 nm in diameter and approx. 18 nm in height. Many researchers observed clear bimodal distribution in the epitaxial growth of Ge on Si [13,14]. Medeiros-Ribeiro et al. [1] showed an energy diagram predicting the existence of two energy minima for the different island shapes at fixed volumes. Ross et al. [14] reported a bimodal distribution attributed to the coarsening process during growth, which leads to a shift in the island size distribution with time. Figure 2a shows the scanning electron microscopy (SEM) image of the sample where Ge islands were grown in the patterned region for 100-nm pit depths. Typically, the holes are of about 120 nm in diameter with a spacing of around 160 nm. It is clear that the islands have nucleated around the periphery of the holes in a circular fashion. This nature of island formation in a circular fashion is present around almost all the holes. Figure 2b shows the SEM micrograph of the grown islands on FIB-patterned substrate with higher pitches (about 500 nm). The preferential circular organization of Ge QDs is more pronounced in this case, as the pitch is large compared to the hole sizes. Therefore, the lateral ordering of islands on patterned substrates has been found to be dependent on the pitch of the holes. Figure 3a,b represents the size distribution of the Ge nano-islands on patterned substrate with 160-and 500-nm pitch, respectively. From Figure 3a,b, it is clear that there is a wide size distribution of Ge islands on patterned substrate. The patterned substrate consists of pits and unpatterned area in between the pits, which leads to a large variation of strain field along the surface. The variation of stain field leads to a wider size distribution.

The transition from 2D layer to 3D island mode for Ge growth occurs randomly on unpatterned substrates, whereas the same occurs preferentially in a circular organization on patterned substrates. It is known that the surface energy of a virgin surface can be increased significantly by ion bombardment. The difference between the chemical potential of a patterned surface and that planar one is described by the change of the surface energy with the surface curvature and the change of the local strain energy induced by the holes [15]. Therefore, when the effect of the stress dominates the surface energy component, the nucleation of dots takes place preferentially on the edges of the holes resulting in circularly ordered islands. The formation of islands in between holes from the residual Ge available on the substrate can be reduced by reducing the pitch of the array, since the mean free path for Ge diffusion is limited [16].

**Figure 4:**
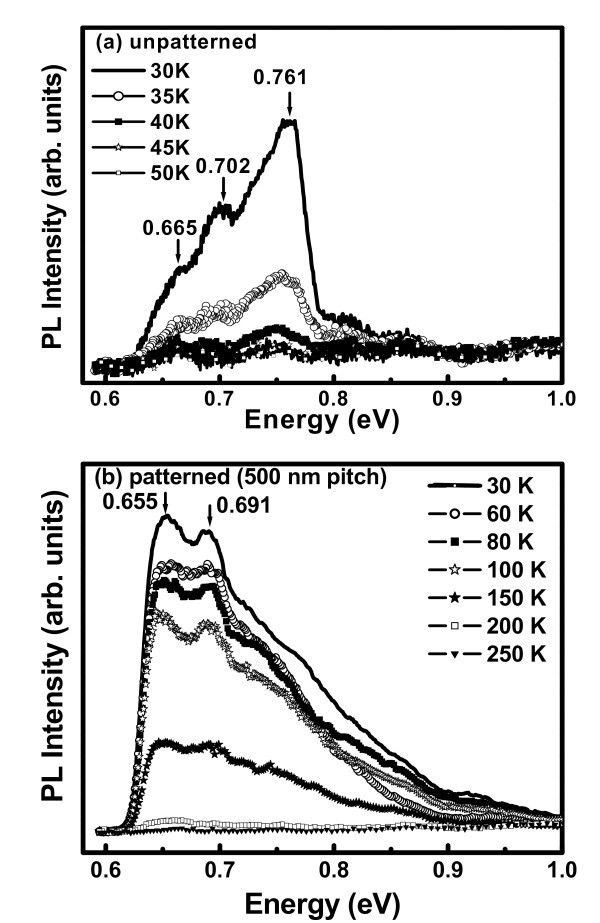
**Temperature-dependent PL from Ge islands grown on (a) unpatterned substrate and (b) patterned substrate (500-nm pitch)**.

**Figure 5:**
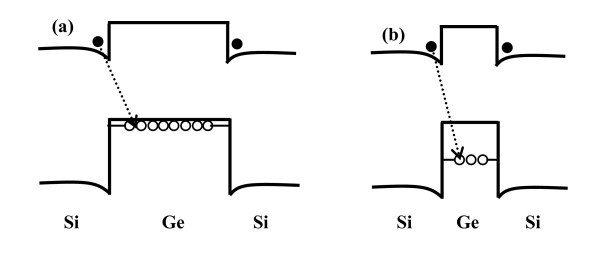
**Schematic band alignment in Ge/Si heterointerface for (a) larger (height 18 nm) and (b) smaller (height 7 nm) islands**.

Figure 4a,b shows the temperature-dependent PL spectra of Ge islands grown on unpatterned and patterned substrates (500-nm pitch sample), respectively. No appreciable PL intensity enhancement was observed for sample with 160-nm pitch over that of unpatterned sample. At a particular temperature (30 K), the PL emission intensity from the highly ordered island (500-nm pitch sample) is one order of magnitude higher than the randomly distributed islands. The details of different PL peak positions and their origins are summarized in Table 1. PL spectra (30 K) from the unpatterned substrate consist of three major peaks at 0.761, 0.702, and 0.665 eV. From Figure 1b, it is clear that islands distribution is nearly bimodal in unpatterned area. The smaller islands have average height approx. 7 nm, whereas the larger ones are approx. 18 nm in height. The observed broad PL peak around 0.761 eV is attributed to the no-phonon (NP) transition of charge carriers localized in and around the smaller islands. Owing to a type-II band alignment, the holes are trapped inside the islands, while the electrons are weakly localized in the strained Si layers around the islands [3]. An asymmetry in the lower energy side of this 0.761 eV peak reveals the existence of TO phonon-assisted transition along with the NP one. The ratio of NP/TO phonon peak intensity is larger in smaller islands because of higher spatial confinement, which leads to the breaking of k-selection rule. The other two peaks located at 0.702 and 0.665 eV, respectively, are identified as the NP and TO phonon lines of larger-sized islands. The separation between NP and TO lines is 37 meV, which is close to the energy of the characteristic Ge-Ge phonons [17]. The energy difference between the NP peaks of smaller and larger islands is about 59 meV. This can be explained by higher confinement energy for smaller islands as PL energy is given by(1)

where *E*_gap,Si _is the bulk Si band gap, Δ*E*_v _is the valence band offset of Ge on Si, and *E*_conf _is the confinement energy which strongly depends upon the height of the nanoislands. Figure 5 schematically represents that the confinement energy (*E*_conf_) for smaller islands (height 7 nm) is larger than that for larger islands (height 18 nm) because of quantum size effect. As all the islands are assumed to have same germanium content, the Δ*E*_v _is same for both types of islands. Hence, the *E*_PL _position is blue shifted for smaller islands versus larger ones grown on unpatterned substrates. Ge islands grown on patterned substrates (500-nm pitch) exhibit PL peaks at 0.691 and 0.655 eV along with a broad luminescence in the range of 0.710-0.850 eV, as shown in Figure 4b. The 0.691 and 0.655 eV PL peaks are assigned to NP and TO phonon-related emissions from ordered islands, respectively, which are around 75 nm in diameter and 17 nm in height. The broad luminescence band in the range of 0.710-0.890 eV observed from the patterned area compared to 0.761 eV PL peak for unpatterned one, is ascribed to the large-size variation and compositional fluctuations within the smaller islands for the former sample. Temperature-dependent PL measurements show that the PL signal from unpatterned sample quenches at a temperature higher than 45 K, whereas it exists up to 200 K for the patterned sample (500-nm pitch). Therefore, the circular ordering of Ge islands plays an important role to sustain the PL signal at a much higher temperature. We have observed enhanced PL form the highly ordered Ge nanoislands (for 500-nm pitch-pattern sample) only. The PL improvement is attributed to lateral coupling between Ge islands. From AFM and SEM images, we have calculated the inter-dot distance among the islands. For unpatterned sample, the average inter-dot distance among the Ge islands is 60 nm, whereas for patterned sample, the average inter-dot distance is 30 nm for 500-nm-pitch sample and is 47 nm for 160-nm pitch pattern. Owing to smaller inter-dot distance and improved circular ordering, the lateral coupling for 500-nm pitch sample is more dominant. Coupling between QDs can occur either (i) via a Coulomb-related interaction, such as the dipole-dipole interaction or the resonant Förster transfer process; or (ii) via a particle tunneling process, whereby the electron or hole or both can move from one dot to the other [18,19]. The actual coupling process that takes place for a given system of QDs primarily depends on the individual dot parameters, the uniformity of the dots, and the inter-dot barrier potential properties, such as the material band gap and thickness [20]. At relatively large inter-dot separations, Coulomb coupling is more likely to occur than single particle tunneling. However, at nanoscale separations, electron/hole tunneling becomes increasingly probable. Clearly, a full configuration model, such as that presented by Bester et al. [20], is necessary for a detailed understanding of such coupling mechanisms. For our case, particle tunneling is the dominant process for lateral coupling due to smaller inter-dot distance. In this case, the particle is a hole due to type-II band alignment of Ge islands on Si. The phonon-assisted hole-tunneling process is confirmed by the PL thermal-quenching activation energy (*E_b _*~ 38 meV). It may be noted that the high PL quenching temperature in this study is only due to lateral ordering without any vertical stacking of islands.

**Table 1:** Summary of different PL peak energies and their origins for both unpatterned and patterned samples

Sample	Island type	Diameter (nm)	Height (nm)	Peak energy (meV)	Origin
Unpatterned	Smaller	65 ± 7	7 ± 1	761	No phonon
	Larger	95 ± 8	18 ± 2	702	No phonon
				665	TO phonon
Patterned (500 nm pitch)	Smaller	40-70	4-10	710-850	No phonon
	Ordered	75 ± 5	17 ± 1	691	No phonon
				655	TO phonon

**Figure 6:**
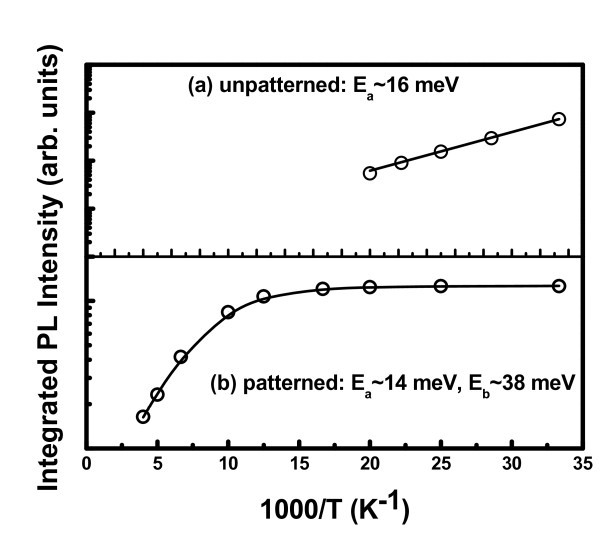
**Temperature-dependent integrated PL intensity of Ge islands grown on (a) unpatterned substrate, and (b) patterned substrate (500-nm pitch)**. Solid lines show the fitting with one and two activation energies for (a) unpatterned and (b) patterned (500-nm pitch) substrates, respectively.

For better understanding of the thermal-quenching mechanism, we have plotted the variation of PL intensity as a function of 1000/T in Figure 6a,b for unpatterned and patterned samples, respectively. The PL intensity temperature dependences is fitted by a standard equation [21](2)

where *I*_PL_(*T*) is the integrated PL intensity at a particular temperature, *C*_1 _and *C*_2 _are two constants, *E_a _*and *E_b _*are the two activation energies for thermal quenching. For unpatterned sample, the best fitting is observed for single thermal activation energy and for patterned sample (500-nm pitch) it is best fitted by two thermal activation energies as shown in Figure 6. From the fitting of PL data for unpatterned sample, we find a single thermal activation energy of *E_a _*~ 16 meV, and for the patterned sample (500-nm pitch) two activation energies of *E_a _*~ 14 meV and *E_b _*~ 38 meV. The low (16 and 14 meV) activation energies are close to the exciton binding energy in SiGe alloys and Si/SiGe superlattices [17,21]. Thus, the above energy can be associated with excitons localized within the compositional fluctuation of the SiGe islands. Owing to type-II band alignment, electron transport in 3D SiGe/Si nanostructures is limited by a small (10-15 meV) conduction band energy barrier [21], as shown in Figure 5. Thus, PL thermal-quenching activation energy of approx. 14-16 meV may be associated with electron migration in SiGe/Si 3D nanoislands. The origin of second activation energy of 38 meV in ordered Ge islands on patterned substrate can be explained in the following way. The hole diffusion in 3D SiGe/Si nanoislands with a high Ge content is controlled by large (> 100 meV) valence band barriers [22]. In this type of system, the electron-hole separation and nonradiative carrier recombination are mainly controlled by hole tunneling between Ge clusters in an ordered array, assisted by the phonon emission and/or absorption [23], with characteristic energy approx. 36 meV. Therefore, the observed 38 meV thermal-quenching activation energy for the ordered islands is close to the Ge/TO phonon energy.

## Conclusions

In conclusion, we have grown the circularly ordered Ge islands by MBE on FIB patterned Si(001) surfaces. The PL spectra obtained from the ordered islands show the existence of the signal up to a temperature as high as 200 K, as compared to 45 K for the control sample. The improvement in PL characteristics in 2D array is attributed to lateral coupling between Ge QDs in the circularly ordered islands. The observed two thermal-quenching activation energies are explained by the competition between phonon-assisted hole tunneling and hole thermoionic emission over the valence band energy barriers at the heterointerfaces.

## Abbreviations

AFM: atomic force microscope; FIB: focused ion beam; HRTEM: high-resolution transmission electron microscopy; IR: infrared; MBE: molecular beam epitaxy; ML: monolayer; NCs: nanocrystals; PC: photocurrent; PL: photoluminescence; QDs: quantum dots; SEM: scanning electron microscopy.

## Competing interests

The authors declare that they have no competing interests.

## Authors' contributions

KD prepared the patterned substrates using FIB. MBE growth of Ge islands was performed by SD, RKS, and SM. SD and SM carried out the temperature-dependent PL measurements. SD and KD performed treatment of experimental data and calculations. SD, KD, and SKR prepared the manuscript initially. SKR, AKR, and AD conceived of the study and participated in its design and coordination. All the authors read and approved the final manuscript.
